# Isolation of a significant fraction of non-phototroph diversity from a desert Biological Soil Crust

**DOI:** 10.3389/fmicb.2015.00277

**Published:** 2015-04-14

**Authors:** Ulisses Nunes da Rocha, Hinsby Cadillo-Quiroz, Ulas Karaoz, Lara Rajeev, Niels Klitgord, Sean Dunn, Viet Truong, Mayra Buenrostro, Benjamin P. Bowen, Ferran Garcia-Pichel, Aindrila Mukhopadhyay, Trent R. Northen, Eoin L. Brodie

**Affiliations:** ^1^Lawrence Berkeley National Laboratory, Earth Sciences DivisionBerkeley, CA, USA; ^2^Quantitative Microbial Ecology Group, Department of Molecular and Cell Physiology, Faculty of Earth and Life Sciences, VU AmsterdamAmsterdam, Netherlands; ^3^Faculty of Genomics, Evolution and Bioinformatics, School of Life Sciences, Arizona State UniversityTucson, AZ, USA; ^4^Lawrence Berkeley National Laboratory, Physical Biosciences DivisionBerkeley, CA, USA; ^5^Lawrence Berkeley National Laboratory, Life Sciences DivisionBerkeley, CA, USA; ^6^Department of Environmental Science, Policy and Management, University of California, BerkeleyBerkeley, CA, USA

**Keywords:** biological soil crusts, culturability, isolation, dryland microbiology, microbial diversity

## Abstract

Biological Soil Crusts (BSCs) are organosedimentary assemblages comprised of microbes and minerals in topsoil of terrestrial environments. BSCs strongly impact soil quality in dryland ecosystems (e.g., soil structure and nutrient yields) due to pioneer species such as *Microcoleus vaginatus*; phototrophs that produce filaments that bind the soil together, and support an array of heterotrophic microorganisms. These microorganisms in turn contribute to soil stability and biogeochemistry of BSCs. Non-cyanobacterial populations of BSCs are less well known than cyanobacterial populations. Therefore, we attempted to isolate a broad range of numerically significant and phylogenetically representative BSC aerobic heterotrophs. Combining simple pre-treatments (hydration of BSCs under dark and light) and isolation strategies (media with varying nutrient availability and protection from oxidative stress) we recovered 402 bacterial and one fungal isolate in axenic culture, which comprised 116 phylotypes (at 97% 16S rRNA gene sequence homology), 115 bacterial and one fungal. Each medium enriched a mostly distinct subset of phylotypes, and cultivated phylotypes varied due to the BSC pre-treatment. The fraction of the total phylotype diversity isolated, weighted by relative abundance in the community, was determined by the overlap between isolate sequences and OTUs reconstructed from metagenome or metatranscriptome reads. Together, more than 8% of relative abundance of OTUs in the metagenome was represented by our isolates, a cultivation efficiency much larger than typically expected from most soils. We conclude that simple cultivation procedures combined with specific pre-treatment of samples afford a significant reduction in the culturability gap, enabling physiological and metabolic assays that rely on ecologically relevant axenic cultures.

## Introduction

Dryland regions, including hyperarid, semiarid, arid, alpine and polar regions, cover approximately one third of the Earth's land surface (Belnap, 2006). Large expanses of these drylands are covered by Biological Soil Crusts (BSCs) (Bowker et al., 2002). BSCs are photosynthetic, diazotrophic communities of bacteria, fungi, algae, lichens and mosses (Belnap, 2006) that have adapted to the infrequent rainfall, high evaporation rates and prolonged UV exposure that limits habitability in these environments. BSCs provide significant ecosystem services within drylands through the aggregation of soil particles that reduces wind and water erosion (Mazor et al., 1996), altering the balance between water run-off and infiltration, thus impacting local hydrologic redistribution (Brotherson and Rushforth, 1983; Kidron and Yair, 1997), and importantly increasing the fertility of soil through nitrogen (N) and carbon (C) fixation (Lange et al., 1994).

BSCs strongly regulate soil quality in dryland ecosystems, are major contributors to soil C-cycling, and represent the significant source of net primary productivity introducing nutrients into soil in many of these ecosystems (Housman et al., 2006). In early successional stages of BSCs, *Microcoleus vaginatus*, a filamentous cyanobacterium, forms a polysaccharide mesh derived from fixed atmospheric C (Mazor et al., 1996). This bed helps further colonization of BSCs by diazotrophic cyanobacteria, other bacteria, fungi, lichens and mosses in a temporal succession (Mazor et al., 1996; Kuske et al., 2012). Hence, *M. vaginatus* is not only the most dominant organism in early successional stages of BSCs (Büdel et al., 2008; Zaady et al., 2010), but a pioneer in the development of this ecosystem.

Better understanding of carbon transformation and flux in early successional stages of BSCs requires knowledge of the direction of carbon flow from the primary producer, *M. vaginatus*, to the non-phototrophic components of the community. Early successional stages of BSCs can harbor a rich community of bacteria (Csotonyi et al., 2010; Steven et al., 2012a; Angel and Conrad, 2013), fungi (Bates and Garcia-Pichel, 2009; Bates et al., 2011; Abed et al., 2013), archaea (Nagy et al., 2005; Soule et al., 2009), protozoa (Bamforth, 2008), and nematodes (Pen-Mouratov et al., 2011). Although it is well known that BSCs can be diverse in composition, the roles of and interactions between community members have not been elucidated. Currently, functional roles of cryptic members of microbial communities can be inferred with different degrees of success using culture-independent approaches (e.g., metagenomics, metatranscriptomics) from soils (Van Elsas et al., 2008), oceans (DeLong et al., 2006), extreme environments (Aliaga Goltsman et al., 2009), and also BSCs (Rajeev et al., 2013). Further, assembly of metagenome short reads mapped to reference databases has helped to reconstruct small and large subunits of the rRNA gene (Miller et al., 2011; Bengtsson et al., 2012). This novel approach has helped to recover the community structure of known and unknown rRNA genes across different domains of life (Nagy et al., 2012; Wrighton et al., 2012) while eliminating biases associated with PCR priming.

Pure cultures of microorganisms are invaluable to validate metagenome-derived hypotheses using simplified well-controlled systems. The highest cultivation efficiency of heterotrophic aerobic bacteria in a dryland ecosystem was achieved in samples from the Atacama Desert. In this ecosystem, up to 0.1% of the bacterial community (by comparison of colony forming units to microscopic cell counts per gram of soil) was cultured in a complex rich medium (Garcia-Pichel et al., 2003; Connon et al., 2007; Lester et al., 2007). However, traditional cultivation techniques often lack key environmental components, such as appropriate concentrations of nutrients, micronutrients and suitable growth matrices, which influence bacterial fitness and culturability. Recent work has demonstrated that improved bacterial culturability can be achieved through the use of more environmentally relevant conditions appropriate to the habitat in question. For example, this was achieved in bulk soil (Janssen et al., 2002; Sait et al., 2002; Schoenborn et al., 2004; Davis et al., 2005; Rocha et al., 2009) and freshwater ecosystems (Bruns et al., 2002, 2003) by reducing nutrient availability, prolonging incubation times and mitigating oxidative stress by the addition of protective agents. Recovery percentages from soils and other environments can thus be increased by simple modifications to existing protocols. However, despite such improvements the overwhelming majority of the bacterial diversity in many ecosystems has remained cryptic so far (Rothschild, 2006) Furthermore, it is not clear what fraction of this diversity is indeed capable of growth and replication. DNA may be extracellular (thus inflating diversity estimates), cells may be permanently injured, and dormant cells require specific conditions and co-factors to induce resuscitation (Dewi Puspita et al., 2012).

Previous attempts to isolate bacteria from BSC, using traditional culture techniques and media, have yielded an important collection of BSC isolates (Gundlapally and Garcia-Pichel, 2006). Different isolates of this library have been described as novel species. They affiliated to Fungi (Bates et al., 2006), Bacteroidetes (Reddy and Garcia-Pichel, 2005, 2013), Actinobacteria (Reddy et al., 2007; Reddy and Garcia-Pichel, 2009), and Proteobacteria (Reddy et al., 2006; Reddy and Garcia-Pichel, 2007, 2015). Phenotyping of these isolates revealed cryptic information regarding their ecology. For example, it was possible to infer presence of pigments (Reddy and Garcia-Pichel, 2005; Bates et al., 2006; Reddy et al., 2006), facultative fermentation strategies (Reddy et al., 2006), psychro- (Reddy and Garcia-Pichel, 2005, 2009), and dessication tolerance (Reddy and Garcia-Pichel, 2007). These examples demonstrate how a larger collection of isolates would improve our understanding of non-photosynthetic organisms in BSC ecosystem functioning.

To attempt to culture a broad range of heterotrophic aerobic bacteria from BSCs we used a polyphasic approach based on: (1) the use of low carbon availability media; (2) the modification of growth media with addition of micronutrients and oxidative stress protective agents; (3) the use of gellan gum as an alternative solidifying agent; and (4) the incubation of samples for prolonged periods (up to 50 days) under an elevated CO_2_ (5%) atmosphere. In addition, prior to isolation, we attempted to resuscitate from stasis different members of the microbial community by pre-incubating BSCs under dark or light conditions to mimic the distinct physiological conditions that occur during a diurnal cycle.

To address which microbes are present and gain information on when they might be activated, we used an amplification- and primer- independent approach, reconstructing the small subunit (SSU) rRNA genes from metagenomic and metatranscriptomic sequence reads using the EMIRGE software (Miller et al., 2011). Finally, we relate the phylogeny of the isolates recovered in this study to those of the reconstructed SSU rRNA gene sequences to determine what fraction of the bacterial diversity was recovered by isolation using these simple modifications to existing cultivation methods.

## Materials and methods

### Sample collection

Petri dishes (6 × 1 cm) were used to core and transport samples of BSCs from the Green Butte Site near Canyonlands National Park (38°42′53.9″N, 109°41′34.6″W, Moab, Utah, USA). All samples were taken in July 2011 from an area of approximately 3 m radius and were 1 cm deep. Details on the sampling site, sampling methodology and storage of the samples prior to the experiment have been previously described (Strauss et al., 2011). Samples were transported air-dry, and stored in dark, under an atmosphere in equilibrium with LiCl desiccant, until experimentation.

### Bacterial isolations

#### Activation of different fractions of the BSC microbial community

Prior to bacterial extraction for bacterial isolation, we attempted to activate two different fractions of the microbial community present in the BSC. To stimulate the microbial community active in day light we added 10 mL of ultrapure sterile water to six Petri-dishes containing BSCs and placed them for 4 h in direct sun light under greenhouse conditions (School of Life Sciences, Arizona State University, Phoenix, AZ, USA). To activate the microbial community stimulated under dark conditions, we added 10 mL of ultrapure water to six other Petri-dishes containing BSC and incubated them in the dark for 5.5 h. These experiments were performed in the greenhouse to mimic conditions from where the BSCs were derived at Moab, UT, (26–28°C) as closely as possible.

#### BSC microbial suspension

After their respective incubation times, we mixed 10 g (wet weight) of the six BSC samples for each of the pre-incubation condition with 190 mL of saline solution (0.95% NaCl) and blended for 1 min at 12,000 rpm (blending was repeated 2 times with a 30 s interval) to release as many bacterial cells as possible. These suspensions were used for dilution plating.

#### Media preparation and plating

For each of the six different BSC samples and both pre-incubation conditions, we used five different solid media to inoculate the BSC suspensions. R2A, a widely used standard medium for cultivation, was modified to different C concentrations from Eaton et al. (2005) to prepare 1/10 R2A, and to prepare 1/20 R2G and 1/100 R2G in which we used gellan gum (Gelrite, Research Products International, Illinois, USA) as an alternative solidifying agent. To prepare 1/10 R2A the following ingredients were dissolved in 1 L of ultrapure water: proteose peptone, 0.05 g; starch, 0.05 g; glucose, 0.05 g; yeast extract, 0.05 g; casein hydrolysate, 0.05 g; dipotassium phosphate, 0.3 g; sodium pyruvate, 0.3 g; magnesium sulfate anhydrous, 0.024 g; agar, 14 g. To prepare 1/20 R2G the following ingredients were dissolved in 1 L of ultrapure water: NaCl, 4.5 g; proteose peptone, 0.025 g; starch, 0.025 g; glucose, 0.025 g; yeast extract, 0.025 g; casein hydrolysate, 0.025 g; dipotassium phosphate, 0.3 g; sodium pyruvate, 0.15 g; magnesium sulfate anhydrous, 0.024 g; gelrite, 12 g. To prepare 1/100 R2G the following ingredients were dissolved in 1 L of ultrapure water: proteose peptone, 0.005 g; starch, 0.005 g; glucose, 0.005 g; yeast extract, 0.00 5g; casein hydrolysate, 0.005 g; dipotassium phosphate, 0.3 g; sodium pyruvate, 0.3 g; magnesium sulfate anhydrous, 0.024 g; gelrite, 12 g. CAT, an oligotrophic medium amended with catalase as oxidative protective agent (Rocha et al., 2009), was modified by using 12 g/L of gelrite as solidifying agent. VXylG, containing many micronutrients and having low carbon availability was prepared as described by Davis et al. (2005). 1/10 R2A, 1/20 R2G, 1/100 R2G, CAT prior to amendment with catalase, and VXylG prior to the addition of the vitamin solutions, were sterilized by autoclaving at 121°C for 20 min. The catalase and vitamin solutions were sterilized by filtration through a 0.2 μm filter (MILLEX GP, Millipore, USA) and added to CAT and VXylG respectively when the medium was cooled to 53°C.

### Colony forming unit (CFU) enumeration, colony selection, and storage of isolates

For CFU enumeration, each of the six BSC suspensions and pre-incubation conditions was serially diluted tenfold in sterile saline (0.95% NaCl), after which (per dilution) 0.1 mL was plated onto the different media. Petri-dishes were kept in their original plastic bags sealed with tape and incubated at 25°C, 18% O_2_ and 5% CO_2_ for 50 days. The increased CO_2_ was used to mimic an aerobic microbial community under high metabolic activity. Colony formation was followed in time by counting the number of CFUs emerging between 3 and 50 days. Comparisons were made between log of CFU counts per gram of dry soil from the different media inoculated with BSCs incubated in the dark or in the light. Differences were calculated by analysis of variance using the “aov” function in the R statistical programming environment, v2.15.1 (R Core Team, 2013). Pairwise comparisons were made using Fisher's Least Significant Difference (LSD) test using the “agricolae” package.

During purification of axenic cultures, we pooled BSC samples per medium and incubation conditions. After 23 days for 1/10 R2A, 35 days for 1/20 R2A, 50 days for 1/100 R2G, and VXylG, and 80 days for CAT, a total of 700 CFUs (~25–50 from each medium per BSC incubation condition) were randomly picked from plates that had received the two highest dilutions of the BSC suspensions. CFUs were streaked to purity three consecutive times on the same medium and allowed to grow to new colonies for up to 4 months. For storage of isolates, cells from axenic cultures were suspended in saline amended with glycerol (final concentration 40%) and stored at −80°C. The experimental design for bacterial isolation is further outlined in Supplementary Figure 1.

### Identification of isolates from BSC by SSU rRNA gene sequencing

For PCR amplification of the SSU rRNA gene, DNA from each of the axenic isolates was extracted using the Bacterial Genomic DNA Isolation Kit (Norgen Biotek Corp., Thorold, ON, Canada) according to the protocol provided by the manufacturer. Later, duplicate 50 μ L PCR reactions containing 10 ng of genomic DNA were prepared as follows: 1X Takara ExTaq Buffer; 2.5 mM; each deoxyribonucleoside triphosphate; 400 nM of each primer, 27F (Lane et al., 1985) and 1492R (Rochelle et al., 1992); and 2.5 U of Takara ExTaq DNA polymerase (Takara Inc, Mountain View, CA, USA). PCR amplifications were performed in a T100 Thermal Cycler (BioRad, USA) with the following thermocycling conditions: one cycle of 94°C for 5 min; 30 cycles at 94°C for 60 s, 55°C for 60 s, 72°C for 90 s; and one cycle at 72°C for 10 min. The PCR products obtained were purified with Agencourt AMPure XP - PCR Purification beads (Brea, CA, USA), and later used for sequencing.

To obtain SSU rRNA gene sequences, PCR products were sequenced from both ends using primers 27F and 1492R at the UC Berkeley DNA Sequencing Facility (Berkeley, CA, USA). The forward and reverse reads for each isolate were assembled using the Geneious® 6.1.2 software (Biomatters Ltd., Auckland, New Zealand). The isolate sequences were then classified using the assignment_taxonomy.py script of the QIIME software package (Caporaso et al., 2010) with the RDP classifier mapping to release 108 of the SILVA database (Quast et al., 2012).

### Metagenomic library construction and sequencing

The DNA used to construct the metagenomic library was extracted from samples collected in the same area as was used for microbial isolation. DNA extraction was performed using bead beating (1.4-mm ceramic spheres, 0.1-mm silica spheres, and one 4-mm glass bead) for cell lysis and phenol chloroform based extraction followed by an AllPrep kit (Qiagen, CA) for nucleic acid purification (Rajeev et al., 2013). Illumina library preparation and sequencing were performed in accordance with the standard protocols of the DOE Joint Genome Institute (Walnut Creek, CA, USA). Briefly, 2 μg of DNA was sheared in 100 μl using the Covaris E210 with the setting of 10% duty cycle, intensity 5, and 200 cycle per burst for 3 min per sample and the fragmented DNA was purified using QIAquick column (Qiagen) according to the manufacturer's instructions. The sheared DNA was end-repaired and A-tailed according to the Illumina standard PE protocol and purified using the MinElute PCR Purification Kit (Qiagen) with a final elution in 12 μl of Buffer EB. After quantification using a Bioanalyzer DNA 1000 chip (Agilent), the fragments were ligated to the Illumina adaptors according to the Illumina standard PE protocol, followed by a purification step of the ligation product using AMPure SPRI beads. The Illumina libraries were quantified using a Bioanalyzer DNA High Sensitivity chip (Agilent) and sequenced on an Illumina HiSeq 2500.

### Metatranscriptomic library construction and sequencing

Previously, we had performed a 3-day wet-up experiment to study the response of *M. vaginatus* to hydration and desiccation in BSCs (Rajeev et al., 2013). The samples used in this study were collected on the same day and from a location within a 3 m radius. To construct the metatranscriptomic libraries we used RNA from the samples 6A (18 h after wet-up, biological replicate A, termed Night time) and 9B (25.5 h after wet-up, biological replicate B, 2.5 h after full lights were on, termed Day time) from the Rajeev et al. (2013) study.

After preparing total RNA as described previously (Rajeev et al., 2013), libraries for Illumina sequencing were prepared using the TruSeq RNA kit (Illumina, USA) according to the instructions of the manufacturer. We used total RNA as our template because our target was rRNA gene sequences. Briefly, the total RNA was chemically fragmented and a first and second strand of cDNA were made. The ends of the cDNA were repaired and the 3′ ends were adenylated. The adenylated cDNA was ligated to the Illumina adapters and library fragments were enriched with 12 cycles of PCR before library purification, quantification and validation. PCR purification was performed using the Agencourt AMPure XP—PCR Purification beads (Brea, CA, USA) according to the procedures described by the manufacturer. To reduce the influence of the dominant community member (*M. vaginatus*) and increase the representation of rRNA from the low abundance species, we treated an aliquot of the total RNA Illumina libraries with a Duplex-Specific thermostable Nuclease (DSN) enzyme (Evrogen, Moscow, Russia) during template re-annealing according to instructions provided by the manufacturers. The DSN treated libraries (hereafter termed DSN-RNA) were PCR enriched for 12 cycles, quantified and validated. The four metatranscriptomic libraries (Day time total-RNA, Day time DSN-RNA, Night time total-RNA, Night time DSN-RNA) were pooled and paired-end sequenced for 151 cycles using an Illumina GAIIx sequencer in the Earth Sciences Division at Lawrence Berkeley National Lab.

### Reconstruction of SSU of rRNA genes from metagenome and metatranscriptome reads

Near-full-length SSU rRNA sequences were reconstructed from Illumina reads with EMIRGE (Miller et al., 2011) using sequence reads from the metagenomic, total-RNA and DSN-RNA metatranscriptomic libraries. We used Trimmomatic (Bolger et al., 2014) as sequence quality trimmer. When below quality level 3, bases were trimmed from the end of the sequences. Paired-end reads where both reads were at least 60 nucleotides in length after trimming were used as inputs. Sixty iterations were run when using emirge.py (metagenome) or emirge_amplicon.py (metatranscriptomes), the maximum length of the sequences was 151 bp, the average insert length was 212 and their standard deviation ranged from 61 to 93 bp. We used all reconstructed sequences for the analysis. The reads were mapped to the release 114NR of the SILVA rRNA gene database (Quast et al., 2012), clustered at 94% similarity, using the tool cluster_fast from the USEARCH software (Edgar, 2010). To the SILVA database 114NR, we added the SSU rRNA gene sequences of the isolates recovered in this study and that of *Microcoleus vaginatus* PCC 9802 (Garcia-Pichel et al., 2001) isolated from the same geographic area as the isolates in this study. Subsequently, we fixed the non-standard characters and constructed a Bowtie index as described by Miller et al. (2011). Chimeric sequences were removed using the reference database mode of the UCHIME algorithm (Edgar et al., 2011); the SILVA database 114NR clustered at 94%, as described above, was used as reference database.

### Comparisons of SSU rRNA genes from isolates and SSU rRNA genes reconstructed from metagenomes and metatranscriptomes

We attempted to determine the fraction of the metagenome and metatranscriptome libraries that were also recovered by cultivation. We binned sequences with more than 97% similarity into the same taxonomic unit. To determine which sequences were more than 97% similar, we used the tool UCLUST from the USEARCH package (Edgar, 2010) where we clustered the SSU rRNA gene sequences of all isolates with those of the SSU rRNA genes reconstructed from metagenomes and metatranscriptomes. Briefly, the fasta files containing the sequences of all isolates were concatenated into the fasta files containing the ribosomal sequences reconstructed by EMIRGE. Sequences were then sorted by length prior to OTU clustering using UCLUST.

We used the R software environment and the package “VennDiagram” version 1.6.0 to determine the intersection of the OTUs isolated across the different media and pre-incubation conditions, and also the intersection between isolate OTUs and OTUs reconstructed from metagenomes and metatranscriptomes. The fractional abundance of the microbial diversity in the metagenomic and metatranscriptomic libraries that also was represented in our isolate pool was denoted as the sum of the relative normalized abundance of the different reconstructed OTUs that were more than 97% similar to the SSU rRNA genes of isolates.

SSU rRNA genes reconstructed from metagenomes and metatranscriptomes were classified using the assign_taxonomy.py script (QIIME, Caporaso et al., 2010). To be able to assign the phylogeny of both Prokaryotes and Eukaryotes we used SILVA database release 108 as a non-default reference database (described in the QIIME tutorials, qiime.org/tutorials/processing_18S_data.html). The isolate with the longest sequence was chosen as cluster representative when comparing only isolates across media or pre-incubation conditions. Phylogenetic trees of SSU rRNA gene sequences of isolates and those reconstructed from metagenomes and metatranscriptomes were made using representatives of these sequences clustered at 97% similarity. The sequences were aligned using the SINA software version 1.2.7 (Pruesse et al., 2012). Jukes-Cantor phylogenetic trees were calculated using the FastTree software (Price et al., 2010) using default parameters. Circular tree layouts were prepared using the online software Interactive Tree of Life (Letunic and Bork, 2011).

### Accession numbers of nucleotide sequences, metagenome, and metatranscriptomes

Sequences of isolate cluster representatives were deposited at EBI under accession numbers LN614590–LN614705 (http://www.ebi.ac.uk/ena/data/view/LN614590-LN614705). The meta-genome library used in this study (total of 134,295,345 paired end reads) is deposited as JGI Project ID 404128 [http://genome.jgi.doe.gov/pages/dynamicOrganismDownload.jsf?organism=LigCruGreenButte]. The four metatranscriptome libraries used in this study are deposited at the EBI European Nucleotide Archive under the project accession number PRJEB7437. The number of paired end reads per library were as follows: Crust_6A_Total, 6,343,659; Crust_6A_DSN, 30,725,320; Crust_9B_Total, 773,360; and Crust_9B_DSN, 10,054,801. As EMIRGE reconstructed SSU rRNA genes are hypothetical sequences, they were not submitted to a public database but are provided as supplementary material (Supplementary_Material_BSC_EMIRGE_outputs.zip).

## Results

### Impact of pre-incubation conditions on isolate recovery

CFUs from the BSCs pre-incubated under dark or light conditions appeared within 2–3 days of incubation (Supplementary Figure 2). Between inoculation and 50 days of incubation, the rate of colony appearance varied by media type and BSC pre-incubation condition. In samples pre-incubated under light, isolate recovery on 1/10 R2A, 1/10 and 1/100 R2G showed similar rates and reached a maximum after 14–21 days, while CAT and VXylG media showed similar initial slopes but extended isolate recovery at a slower rate from days 14–50. Isolate recovery trajectories from BSCs pre-incubated under dark conditions showed divergent patterns from those under light conditions with 1/100 R2G showing a slower rate of accumulation.

**Figure 1:**
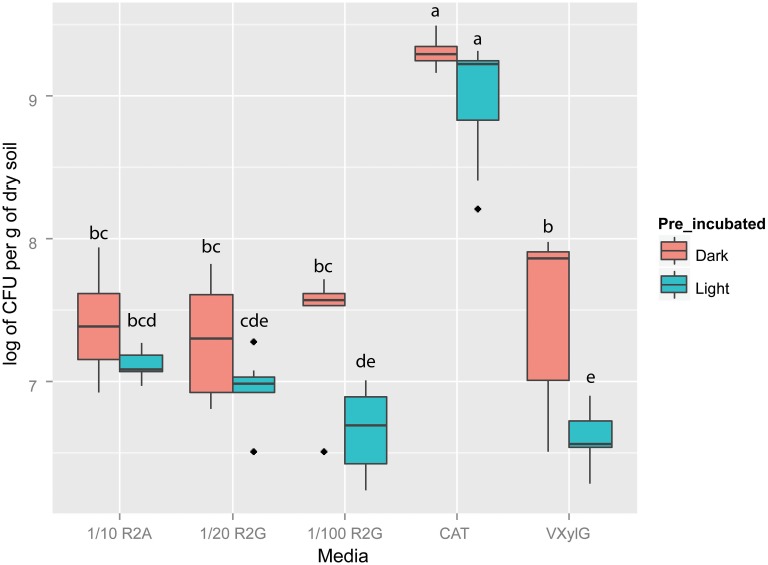
**Boxplot of colony forming units on different media (i.e., 1/10 R2A, 1/20 R2G, 1/100 R2G CAT, and VXylG) after 50 days incubation**. Vertical bars represent standard deviations of the mean. Top and lower lines of boxes represent, respectively, 75th and 25th percentiles. Horizontal bars represent the medians. Points outside the boxes represent outliers. Light/Dark indicate plates counted from samples incubated, respectively, in the light and in the dark. Boxes with same letters are not statistically different according to *t*-test (*p* > 0.05).

After 50 days of incubation, there was a general trend of more CFUs per gram of dry soil being obtained in samples pre-incubated in the dark relative to those pre-incubated in the light (Figure 1). Fisher's LSD test showed CAT plates incubated both under dark and light conditions had the highest CFU number (Figure 1). In 1/10 R2A and 1/20 R2G, we observed no significant difference between media inoculated with BSCs pre-incubated in the dark or in the light in samples incubated (*p* > 0.05). Both 1/100 R2G and VXylG showed higher CFU numbers in plates incubated in the dark (*p* < 0.05). The lowest averages of CFUs were observed in 1/20 R2G, 1/100 R2G, and VXylG incubated under light conditions.

We attempted to purify 700 colonies from all media. From those, 580 were successfully purified to single colonies, and 524 isolates were successfully streaked to purity after three continuous passes. For media inoculated with BSC samples pre-incubated in the light, 34, 31, 55, 12, and 21 isolates were recovered on 1/10 R2A, 1/20 R2G, 1/100 R2G, CAT, and VXylG respectively (*n* = 153). For media inoculated with BSC samples pre-incubated in the dark, 73, 52, 91, 5, 28 isolates were recovered on 1/10 R2A, 1/20 R2G, 1/100 R2G, CAT, and VXylG respectively (*n* = 249).

### Different media recover phylogeneticaly distinct organisms

The sequences of the SSU rRNA gene of 401 isolates from all five media were affiliated with four bacterial phyla. Isolate D2B05, recovered from 1/20 R2G, was classified as a fungal eukaryote within the Agaricomycotina subdivision (Table 1). Representatives of Actinobacteria and Proteobacteria (mostly Alphaproteobacteria) were obtained from all media. Firmicutes were recovered from 1/20 and 1/100 R2G only and Bacteroidetes from VXylG only. Overall the majority of isolates were affiliated with the Actinomycetales order (Table 1).

**Table 1:** **Taxonomic distribution of isolates from the early successional stage desert Biological Soil Crust from Moab, UT**.

**Domain**	**Phylum**	**Class**	**Order**	**Family**	**Dark^a^**	**Light^b^**
Bacteria	Actinobacteria	Actinobacteria	Actinobacteridae	Actinomycetales	126	192
			Rubrobacteridae	Solirubrobacterales	5	4
	Bacteroidetes	Sphingobacteria	Sphingobacteriales	Cytophagaceae	-	**4**
	Firmicutes	Bacilli	Bacillales	Bacillaceae	8	9
	Proteobacteria	Alphaproteobacteria	Rhizobiales	Beijerinchiaceae	-	**1**
				Bradyrhizobiaceae	4	12
				Methylobacteriaceae	1	1
			Rhodospirillales	Acetobacteraceae	7	25
		Betaproteobacteria	Burkholderiales	Comamonadaceae	-	**1**
		Gammaproteobacteria	Pseudomonadales	Moraxellaceae	**1**	-
Eukaryota	Fungi	Dikarya	Basidiomycota	Agaricomycotina	**1**	-
Total					153	249

a Isolates recovered from Biological Soil Crust samples incubated in the Dark.

b Isolates recovered from Biological Soil Crust samples incubated in the Light.

Numbers in bold indicate taxa found in only one pre-incubation condition.

**Figure 2:**
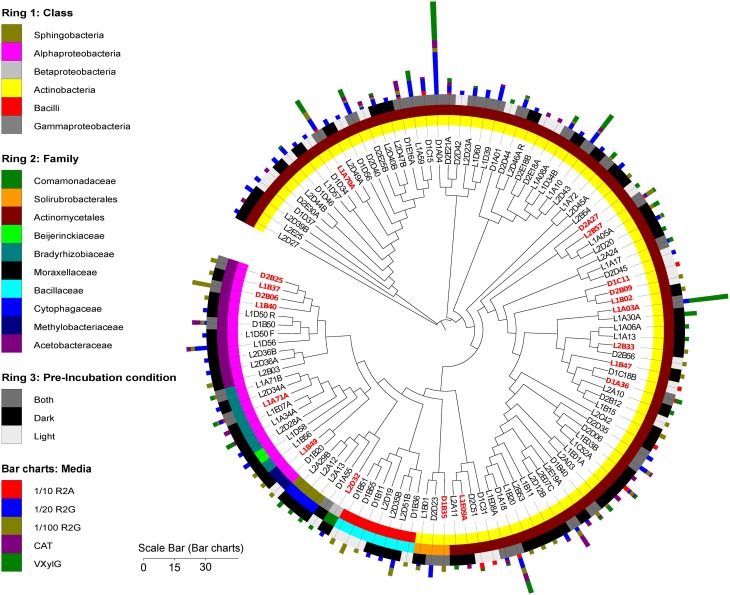
**Phylogenetic tree with representative sequences of the bacterial isolates clustered at 97% similarity**. Leaf labels indicate representative sequence. Rings, from the inner to the outside circles, represent: Ring 1, taxonomy (class level) of cluster representative isolate; Ring 2, taxonomy (family level) of cluster representative isolate; Ring 3, pre-incubation condition of Biological Soil Crust samples prior bacterial isolation procedures. Bar charts, the different media from where the isolates were recovered. Size of the bars indicates the number of isolates in each of the clusters. Red and bold leaf labels indicate which representative sequences clustered at 97% similarity with OTUs reconstructed from the metagenome or metatranscriptome libraries. Scale bar indicates the number of isolates per representative sequence.

Clustering of the isolates at 97% 16S rRNA gene sequence homology yielded a total of 116 clusters (115 bacterial and one fungal) among the 402 isolates (Figure 2). Notably, only two clusters were detected across all five media (Figure 2). These clusters were represented by isolates L1C52A and D1A18 (Figure 2) and were affiliated with the genera *Williamsia* and *Microbacterium* respectively. The cluster with the largest number of isolates was affiliated with *Arthrobacter globiformis* (accession number JF439618, 99% similar to representative isolate D1A04).

**Figure 3:**
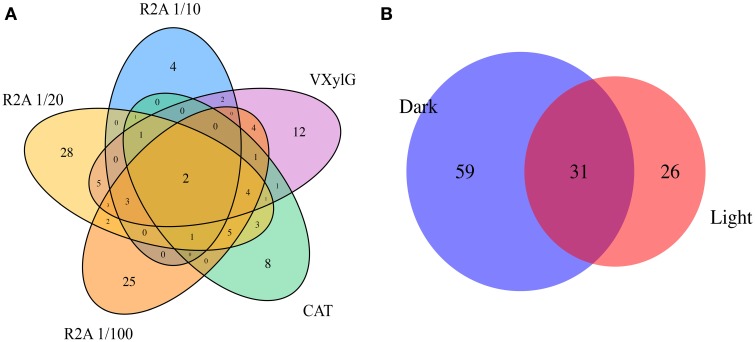
**Venn diagrams showing the intersection of isolate clusters found in (A) the different media (i.e., 1/10 R2A, 1/20 R2G, 1/100 R2G, CAT, and VXylG) and (B) pre-incubation conditions (Dark and Light)**.

The majority of the bacterial clusters (77 out of 115) were recovered from a single medium (Figure 3A), while the remaining were distributed over 2–5 media, indicating that each medium selected for distinct fractions of the BSC microbial community. Similarly, pre-incubation conditions selected for a subset of the microbial community with only about 27% (31 of 115) being derived from both light and dark pre-incubations (Figure 3B). For the complete distribution of isolate clusters according to their media and pre-incubation conditions, refer to Supplementary Table 1.

### Only *M. vaginatus* was detected across all metagenomic and metatranscriptomic libraries

In order to compare the diversity of the recovered isolates to the original BSC population, we analyzed rRNA genes using a PCR-independent approach to eliminate PCR primer bias. After removal of chimeric sequences (23.1% of the total), reconstruction of SSU rRNA genes using EMIRGE analysis of shotgun metagenomic reads from this BSC resulted in 251 full-length sequence clusters at 97% similarity. Estimates of sequence abundances using coverage information suggested dominance of Bacteria with a minor fraction of Archaea (99.4 and 0.6% respectively, Supplementary Table 2). As expected, the dominant taxon was classified as *M. vaginatus* at over 41% abundance. The most abundant non-cyanobacterial phylotypes were represented by members of the Actinomycetales (18.2%) and the Alphaproteobacteria (13.3%).

Shotgun metatranscriptomes were prepared using RNA obtained from samples taken during light and dark sampling time points from a BSC wet-up experiment that has been previously described (Rajeev et al., 2013). As we expected the dominance of *M. vaginatus* to inhibit the detection of less abundant bacterial taxa, we used a library normalization approach (duplex-specific thermostable nuclease enzymatic digestion, termed DSN-RNA) typically employed to reduce the influence of abundant housekeeping transcripts in mRNA analysis (Bogdanova et al., 2009) and we compared this to a standard shotgun RNA library derived from the same material (total-RNA). After removal of chimeric sequences (approximately 9.3% of the total), Metatranscriptomes obtained from light (day time) samples had a strong dominance of *M. vaginatus* (69%) and total sequences for cyanobacteria comprised more than 82% of the predicted rRNA gene abundances. This contrasts with the dark (night time) sample where cyanobacteria abundances were approximately 50%. As the proportion of cyanobacteria RNA phylotypes declined at night, numerous bacterial groups became detectable (Supplementary Table 2). After removal of chimeric sequences (approximately 13.7% of the total), over 4700 and 3800 reconstructed SSU rRNA gene OTUs were derived from the dark (night) sampling total-RNA and DSN-RNA respectively (Supplementary Table 2). These OTUs were distributed between Archaea, Bacteria and Eukarya, with again most sequences being affiliated with the Bacteria, although the abundance of both Archaea and Eukarya were proportionally greater in the night time DSN-RNA library compared to the total-RNA library. The libraries derived from day-time sampling yielded substantially fewer numbers of OTUs, however DSN treatment resulted in a four-fold increase in OTU numbers suggesting the low estimate of diversity was due to cyanobacterial dominance. Analysis of the *M. vaginatus* proportion of the sequences shows an abundance of approximately 25% in the total-RNA library declining to less than 10% after DSN treatment and the proportion of non-phototrophic bacteria increasing from 17% to over 70% (Table 2).

**Table 2:** **Relative abundance (percentage) of rRNA OTUs and overlap at 97% similarity between metagenome (MG) and metatranscriptome (MT) reconstructed OTUs and isolated bacteria**.

**Sequence type**	**Sample**	**Treatment^a^**	**Library prep.^b^**	**Number of EMIRGE OTUs**	**Relative Abundance^c^ (Domains)**			**Relative Abundance (Bacteria)**			**Relative abundance of isolates with >97 similarity to MG/MT reconstructed sequences**		
					**Bacteria (%)**	**Archaea (%)**	**Eukaryota (%)**	***M. vaginatus* (%)**	**Cyanobacteria (non *M. vaginatus*) (%)**	**Non-cyanobacteria (%)**	**Number**	**Total Bacteria (%)**	**Non-phototrophic Bacteria (%)**
Metagenome	MTG	Prior wet-up	Total DNA	251	99.43	0.57	BDL	41.34	6.69	51.97	8	4.28%	8.23%
Metatranscriptome	6	Night time (18 h)	Total RNA	4708	98.03	0.26	1.71%	16.96	34.80	48.25	16	1.55%	3.21%
			DSN treated	3829	92.82	0.58	6.60%	3.23	6.41	90.36	12	2.98%	3.30%
	9	Day time (25.5 h)	Total RNA	50	99.75	0.25	BDL	24.59	58.15	17.26	2	0.75%	4.37%
			DSN treated	213	99.92	0.08	BDL	9.67	19.54	70.79	1	BDL	BDL

a The metagenomic library was prepared with DNA extracted from samples Prior wet-up, and the metatranscriptomic libraries were prepared with RNA extracted 18 h (night time) or 25.5 h (day time) after wet-up.

b RNA used for library preparation. The libraries were prepared with total cDNA (Total RNA) or Double Strand Specific Nuclease (DSN) treated cDNA to reduce the influence of dominant organisms (DSN treated).

c BDL, below detection limit.

Comparisons between the different sequences reconstructed by EMIRGE from the metagenome and metatranscriptome libraries demonstrated that only *M. vaginatus* was detected across all sequencing libraries (Supplementary Figure 3). From the 251 OTUs detected in the metagenome, 38 were found in at least one of the metatranscriptomic libraries (Supplementary Figure 3).

### Representation of isolates within the BSC microbial community: comparison to metagenome and contrasting metatranscriptomes

To determine what fraction of the total phylotype diversity (OTUs weighted by relative abundance in the community) in our BSC metagenome and metatranscriptomes was recovered as isolates, we determined the overlap between isolate sequences and OTUs derived from metagenome or metatranscriptome sequences (Supplementary Figure 4). Not including *M. vaginatus* which was previously isolated from these crusts, 7 OTUs were shared between the isolates and the metagenome reconstructed sequences. When considering the proportional abundances of these metagenome OTUs (excluding *M. vaginatus*), at 97% homology these isolates represented approximately 8% of the bacterial relative abundance (Table 2). Similarly, 16 and 12 isolates overlapped with sequences from the dark (night time) total-RNA and DSN-RNA metatranscriptomes respectively, representing approximately 3.2% of the community relative abundance. The 16S rRNA gene of two isolates clustered at 97% homology with OTUs reconstructed from the light (day time) total-RNA metatranscriptome. These two OTUs comprised 4.4% of the relative abundance observed for that metatranscriptomic library. Supplementary Table 3 shows the phylogenetic affiliation and relative abundance of OTUs with isolated representatives.

## Discussion

Here, we report the first attempt to assess what fraction of the aerobic heterotrophic bacterial diversity of an early successional stage biological soil crust could be cultivated by simple plating under aerobic conditions. To this end, we isolated over 400 bacteria using five different media formulations with varying nutrient concentration, solidifying agents, presence of oxidative-protective agents and micronutrients. We developed a number of reference sequence datasets to place the isolate diversity in the context of BSC diversity under distinct physiological conditions (dry, wet, dark, light) by using a primer- and amplification-independent method (EMIRGE), to reconstruct rRNA gene sequences from shotgun metagenomes and metatranscriptomes.

Over the past 15 years, numerous strategies have been developed to obtain axenic or mixed cultures of many soil microorganisms previously classified as unculturable (Janssen et al., 2002; Sait et al., 2002; Bruns et al., 2003; Schoenborn et al., 2004; Davis et al., 2005; Rocha et al., 2009). Once isolated, it is challenging to place organisms into a useful environmental context and most studies have compared the yield of isolates in terms of numbers relative to direct counts of soil microorganisms; it is from these types of comparisons that the classical descriptions of the proportion of culturable organisms and the “great plate count anomaly” are derived (Suzuki et al., 1997; Bruns et al., 2002, 2003; Connon and Giovannoni, 2002; Janssen et al., 2002; Sait et al., 2002; Ellis et al., 2003; Tamaki et al., 2005; Connon et al., 2007; Lester et al., 2007; Shivaji et al., 2011). In contrast, few attempts have been made to assess the fraction of microbial diversity that can be cultured (Kämpfer et al., 1996; Suzuki et al., 1997; Tamaki et al., 2005; Rocha et al., 2009; Shivaji et al., 2011; Colin et al., 2013) and their numerical importance and most of these studies have characterized diversity using cloning and sequencing to a limited extent. Here, our use of multiple media, different pre-incubation conditions and extended incubation times was designed to improve the recovery of a more diverse range of bacterial heterotrophs from BSCs. Similarly, our use of BSC samples taken under distinct physiological conditions (dry, wet, dark, light) to reconstruct their phylogenetic composition using non-amplification based methods, was designed to improve our knowledge of the true diversity in these systems and to provide an appropriate context to place cultivated taxa.

As expected, the appearance of visible CFUs differed by both media type and pre-incubation condition. In general, media with lower nutrient concentrations and oxidative stress protectants selected for more organisms with slower growth rates as evidenced by the extended recovery of isolates out to 50 days. Similarly, Davis et al. (2005) demonstrated that in media with high nutrient concentrations, isolates ceased to appear 2–4 weeks after inoculation. When comparing the recovery of isolates across media types, it was significant that only the CAT medium, containing low nutrient concentrations and amended with catalase as a protectant against oxidative stress, showed significantly more recovered CFUs than other media. Previous efforts to retrieve culturable isolates from BSCs obtained CFUs of approximately 6.8 log units per g of BSC (Garcia-Pichel et al., 2003) which was similar to the range reported here, with the exception of CFUs recovered on CAT media that were approximately 1.5 log units greater. Although in a rhizosphere system, the use of the CAT medium also resulted in higher CFU counts compared with 1/10 R2A (Rocha et al., 2009), for bacteria inhabiting a BSC this was somewhat surprising as given its predominantly oxidizing nature (with the exception of dark active periods) one might expect traits associated with oxidative stress protection to be selected for in many individuals (Wang and Sheng, 2011; Steven et al., 2012b; Rajeev et al., 2013; Reddy and Garcia-Pichel, 2013).

Although here we have reported the appearance of visible colonies, it has been observed that micro-colonies (below 250 μm diameter) may comprise a large fraction (on average 48%) of CFUs from rhizosphere soils (Rocha et al., 2009) and over 75% from gardening soil (Watve et al., 2000) and that micro-colonies may continue to emerge over 12–24 weeks post-inoculation (Davis et al., 2011). Therefore, we acknowledge that our recovered isolates likely missed organisms with this growth property. However, as our overall goal was the recovery of organisms to enable hypothesis testing related to physiological roles and functions inferred from metagenomic approaches, obtaining a broad diversity of organisms that were also numerically abundant in our BSC was the objective.

The rate of isolate recovery for a specific medium also varied somewhat based on the pre-incubation condition used, suggesting that a different subset of the bacterial population may have been activated due to this treatment. In fact, of the 116 (115 bacterial and one fungal) OTUs represented by the isolates, only 31 were in common between the dark and light pre-incubated samples (Figure 3B). One significant example of this differential recovery is the Bacteroidetes members (four *Hymenobacter* species) that were only recovered from the dark incubated samples. Relatives of these isolates have been recovered from similar crusts in the Colorado Plateau previously (Reddy and Garcia-Pichel, 2013). We only observed differences in CFU numbers between plates pre-incubated under light and those pre-incubated in the dark in 1/100 R2G and VXylG. The current study was designed to yield higher diversity of aerobic heterotrophs, however anaerobes and chemoautotrophic physiologies are also likely important in these systems and further studies should target their isolation and pre-activation.

Analysis of the overlap in isolate recovery across all media (at 97% homology, Figure 3A) shows the majority of the clusters (>66%) were recovered from only a single type of media. The only two isolates recovered in all five different media were affiliated with species belonging to the Actinomycetes *Williamsia* spp. and *Microbacterium* sp.. Representatives of *Williamsia* are known to inhabit arid environments such as Antarctic desert soils (Guerrero et al., 2014) and have been previously isolated from BSCs (Gundlapally and Garcia-Pichel, 2006) as have *Microbacterium* sp. (Gundlapally and Garcia-Pichel, 2006). The diversity of isolates recovered in our study expanded that observed by Garcia-Pichel et al. (2003) by isolating not only members of Methylobacteriaceae, Bacillaceae, and Actinomycetales, but also Comamonadaceae, Solirubrobacterales, Beijerinckiaceae, Bradyrhizobiaceae, Moraxellaceae, Cytophagaceae and Acetobacteraceae. The only group isolated by Garcia-Pichel et al. (2003) not found in this study was Caulobacteraceae. Another attempt to isolate a broad range of isolates from the crust managed to isolate 34 different species (grouped at 97% similarity) from the same area where our samples were collected (Gundlapally and Garcia-Pichel, 2006). None of these isolates showed more than 97% similarity to the isolates recovered in the current study (data not shown), supporting the hypothesis that each medium/incubation condition or even different isolation attempts will yield a novel set of species. Alternatives to this hypothesis would be that: due to the limited number of isolates, each sample has not reached saturation in isolates recovered in each medium and hence phylogeny in each medium and pre-incubation condition look different; or, every piece is indeed different and just the major players are the same.

A total of 251 different SSU rRNA gene OTUs were obtained by EMIRGE (Miller et al., 2011) reconstruction from metagenome reads. A previous study including a shotgun metagenome of dryland soil microbial communities detected the presence of Cyanobacteria, Proteobacteria, Actinobacteria, Chloroflexi Acidobacteria, Planctomycetes, OP10 and Firmicutes in both early and late successional BSC developmental stages in the Nevada Desert (Steven et al., 2012a). In our study, with the exception of OP10, we detected all phyla observed by Steven et al. (2012a). Further, we also detected Archaea, Gemmatimonadetes, Verrucomicrobia, and Fungi. Another study in arid and hyperarid BSC (Angel and Conrad, 2013) did not detect the presence of Chloroflexi, Gemmatimonadetes or Verrucomicrobia among the detected bacterial groups in dry crust. Differences observed between our study and that of Steven et al. (2012a) and Angel and Conrad (2013) may be related to the different geographical positions of the BSC tested in both studies, as well as differences caused by barcoding, PCR amplification and high-throughput sequencing.

We constructed four metatranscriptomic libraries from this BSC to determine the sequences of organisms with intact ribosomes under two contrasting physiological conditions (dark and light). Two of these libraries were constructed with total RNA (Total-RNA) and these libraries were split and then treated with a duplex-specific nuclease (DSN) to enrich the detection of low abundance transcripts (Yi et al., 2011), generating a second set of libraries termed DSN-RNA. We hypothesized that DSN treatment would improve our representation of the true diversity of BSCs by reducing the masking effect of dominant cyanobacteria. EMIRGE reconstruction of SSU rRNA genes from the total-RNA library sampled under light conditions confirmed this hypothesis where *M. vaginatus* sequence relative abundance was reduced from ~70 to ~24% and Alphaproteobacteria increased from 5% to about 40% (Supplementary Table 1). Concurrently, detected OTUs increased from 56 to 213 with certain broad groups such as the Actinomycetales going from undetectable to prominent. In contrast to this, DSN treatment of the BSC metatranscriptomes sampled under dark conditions also showed a shift in dominance away from *M. vaginatus* (44 to 6%) to Actinomycetales (~8 to 19%). Further, the impact of sampling in the dark alone (*M. vaginatus* rRNA abundance declined from ~70 to 44%) was sufficient to uncover substantially greater diversity compared to that found in samples taken under light conditions. Although changing the detectable species diversity, DSN treatment did not always result in an increase in OTUs detected. This highlights the value of using natural shifts in metabolic activity, such as those observed between light and dark conditions in these samples (Rajeev et al., 2013) to improve representation of microbial groups in these systems. While shifts in rRNA relative abundance may not be directly correlated with changes in activity (Blazewicz et al., 2013), we observed shifts in relative rRNA abundance between the light and dark samples (separated by only 3 h) with a sharp decline in cyanobacteria. These abrupt changes clearly have biological relevance and suggest that organisms in BSCs rapidly shift between metabolic states and alter cellular ribosomal content within hours.

After establishing an improved view of the true diversity of these BSCs, we calculated the relative cultivation efficiency relative to OTUs uncovered using the metagenome and metatranscriptome analyses. Overall, our isolates encompassed just over 8% of the non-phototrophic bacterial abundance in the metagenome. When accounting for the presence of *M. vaginatus* (Garcia-Pichel et al., 2001), as estimated using EMIRGE reconstruction (Supplementary Table 3), more than 45% of the OTUs in this BSC metagenome have been represented by culture-dependent techniques. We also hypothesized that the best concordance between our cultured isolates and the various windows of microbial diversity estimated here would be achieved when comparing to metatranscriptomes rather than metagenomes as organisms detected due to their rRNA are more likely to be metabolically active. Overall, 33% of our isolated OTUs matched closely (97%) to OTUs detected by sequence reconstruction. The majority of matches corresponded to sequences from the dark metatranscriptome, and only few matching those from the light metatranscriptome.

The cultivation efficiency in desert soils is reported between 0.0007 and 0.1%, calculated by comparing CFU counts with those visualized by microscopy (Connon et al., 2007; Lester et al., 2007). A previous attempt to isolate aerobic copiotrophs from BSC samples from the same area where we acquired our samples, resulted in 0.51% cultivation efficiency, again based on comparison of CFUs to microscopic counts (Garcia-Pichel et al., 2003). However, the units and parameters found in the previous studies may not be comparable to the ones reported here.

In the current study, we recovered up to 8% of the microbial diversity detected in these BSC communities. The detection of diversity, and thus estimates of recovery are clearly impacted by the structure (e.g., evenness) of the community sampled and also the depth of sequencing performed. Due to these interacting factors, EMIRGE reconstruction may not capture sequences that exist in a long tail of low abundance organisms. Additionally, in our study, only 15% of the ribosomal genes reconstructed from the metagenome were also found in the metatranscriptomic libraries, suggesting that many detected OTUs may originate from dormant or dead organisms or possibly extracellular DNA—making a true estimate of the composition of a soil microbiome problematic. Nevertheless, to the best of our knowledge, ours is the first study to attempt cultivation efficiency calculation in terms of OTU recovery without the influence of PCR amplification bias.

## Conclusions

In the age of genomics, the value of isolated microorganisms is frequently overlooked, yet all observations from genomics remain hypotheses until validated via biochemical or physiological assays either *in vitro* or *in vivo*. We combined amplification independent analysis of microbial populations with media of varying composition to recover bacteria that were differentially resuscitated from stasis through pre-incubation. We recovered 402 bacterial and one fungal isolate in axenic culture, comprising 116 OTUs (115 bacterial and one fungal). We found that each medium recovered mostly distinct OTUs and that our view of the true diversity was significantly impacted by the physiological state of the BSC when sampled, with dominant organisms frequently obscuring our detection of less abundant members. We related the recovered isolates to the fraction of biomass (estimated abundance) of common OTUs in the BSC and found that, not including the dominant cultured phototroph, more than 8% of the relative abundance was represented by our isolates. Although not completely closing the culturability gap in BSCs, our study indicates that it is possible to reduce this gap using simple cultivation procedures.

### Conflict of interest statement

The authors declare that the research was conducted in the absence of any commercial or financial relationships that could be construed as a potential conflict of interest.
