# Supplementary material for: Isolation of a significant fraction of non-phototroph diversity from a desert Biological Soil Crust
Source: Front Microbiol. 2015 Apr 14;6:277. doi: 10.3389/fmicb.2015.00277 (PMC4396413; doi:10.3389/fmicb.2015.00277)
Supplement: Supplementary file 2 [file Table2.PDF]

Supplementary Table 2. Relative abundance (percentage) of EMIRGE outputs reconstructed from the different libraries.

Taxa	Metagenome		Metatranscriptome - Night time				Metatranscriptome - Day time			
			Total RNA[a]		DSN Treated[b]		Total RNA		DSN Treated	
	Abundance (%)	OTUs	Abundance (%)	OTUs	Abundance (%)	OTUs	Abundance (%)	OTUs	Abundance (%)	OTUs
Archaea	0.6	2	0.3	2	0.6	2	0.3	1	0.1	1
Acidobacteria	1.5	16	1.8	480	5.8	428	3.4	7	0.1	1
Actinobacteria_Acidimicrobiales	0.9	9	1.0	180	2.7	171	3.0	5	0.2	2
Actinobacteria_Actinomycetales	**18.2**[c]	50	**8.7**	496	**18.9**	459	BDL	0	2.7	35
Actinobacteria_others	BDL	0	0.3	42	0.6	34	BDL	0	BDL	0
Actinobacteria_Rubrobacteridae	7.2	26	4.2	219	**10.8**	206	BDL	0	1.2	12
Bacteroidetes	4.4	23	1.7	202	1.7	156	0.5	2	0.7	9
Chloroflexi	1.5	8	2.2	205	3.3	148	BDL	0	0.6	12
Cyanobacteria_Microcoleus	**44.3**	7	**36.8**	83	6.4	49	**69.3**	12	**23.6**	30
Cyanobacteria_others	3.5	12	**13.9**	143	2.5	63	**13.2**	13	5.6	21
Firmicutes	1.6	9	4.7	146	5.0	86	1.6	3	1.0	8
Gemmatimonadetes	BDL	0	2.0	108	1.1	75	1.6	2	1.0	5
Planctomycetes	1.0	7	4.1	477	**11.7**	442	0.3	1	0.5	8
Proteobacteria_Alphaproteobacteria	**13.3**	62	8.1	765	10.4	538	**5.1**	8	**39.0**	48
Proteobacteria_Betaproteobacteria	0.4	6	1.5	251	2.2	169	1.5	1	1.1	11
Proteobacteria_Deltaproteobacteria	0.4	3	2.3	238	5.1	150	BDL	0	0.1	2
Proteobacteria_Gammaproteobacteria	BDL[d]	0	0.2	59	0.4	51	BDL	0	BDL	0
Proteobacteria_others	BDL	0	0.1	35	0.3	29	BDL	0		0
Verrucomicrobia	0.1	1	0.7	146	1.7	193	BDL	0	0.1	1
Bacteria_other	1.2	10	3.4	258	2.3	192	0.1	1	**22.6**	7
Fungi	BDL	0	1.3	80	5.3	80	BDL	0	BDL	0
Nematoda	BDL	0	0.0	9	0.2	17	BDL	0	BDL	0
Plantae	BDL	0	0.1	18	0.2	19	BDL	0	BDL	0

Protozoa	BDL	0	0.1	24	0.3	21	BDL	0	BDL	0
Eukaryotes_other	BDL	0	0.2	44	0.7	51	BDL	0	BDL	0
Total		251		4710		3829		56		213

[a] Total RNA, metatranscriptomic libraries constructed with total cDNA.
[b] DSN Treated, metatranscriptomic libraries constructed with cDNA treated with double strand nuclease.
[c] Numbers in bold represent the three most abundant taxa per library.
[d] BDL, below detection limit
