# Supplementary material for: Isolation of a significant fraction of non-phototroph diversity from a desert Biological Soil Crust
Source: Front Microbiol. 2015 Apr 14;6:277. doi: 10.3389/fmicb.2015.00277 (PMC4396413; doi:10.3389/fmicb.2015.00277)
Supplement: Supplementary file 5 [file Image1.PDF]

Supplementary Figure 1

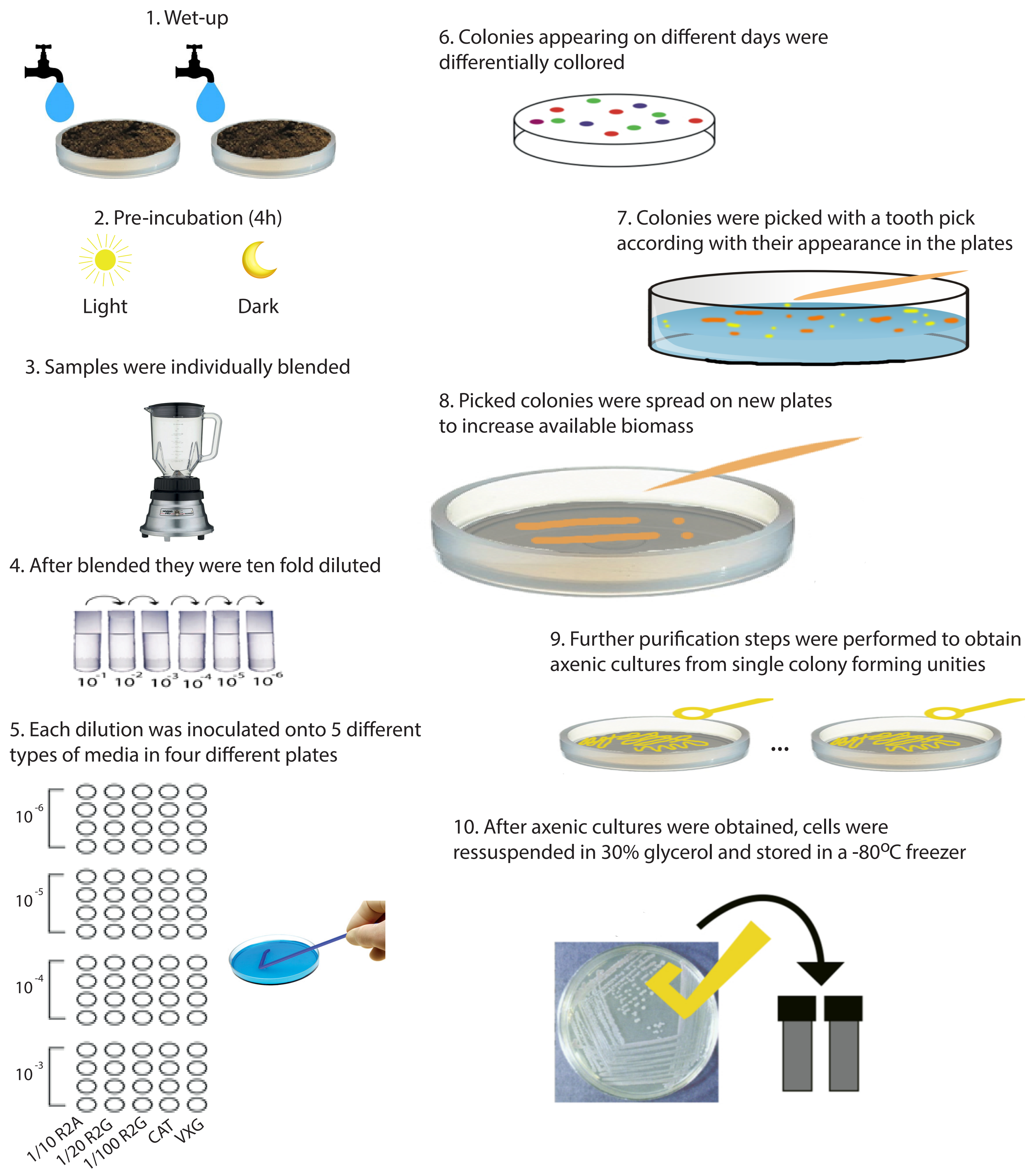

Figure legend. Scheme representing isolation procedures (for description, see text)
