# Supplementary material for: Isolation of a significant fraction of non-phototroph diversity from a desert Biological Soil Crust
Source: Front Microbiol. 2015 Apr 14;6:277. doi: 10.3389/fmicb.2015.00277 (PMC4396413; doi:10.3389/fmicb.2015.00277)
Supplement: Supplementary file 6 [file Image2.PDF]

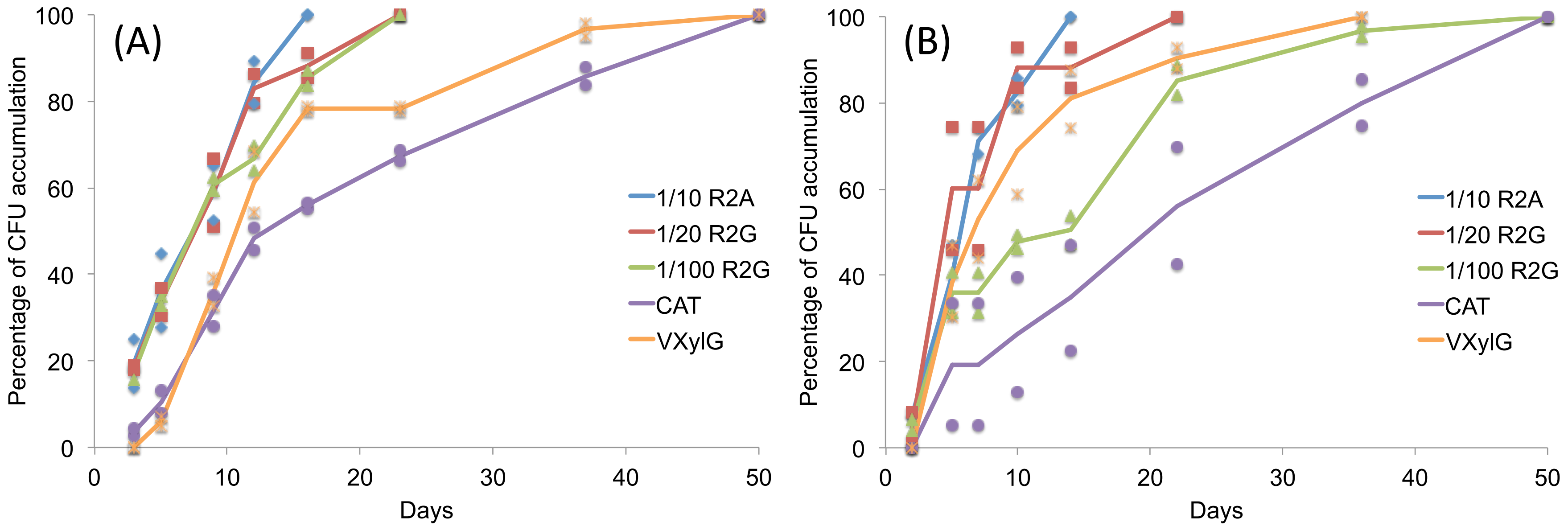

Figure legend. Colony appearance over time on different media. Symbols colored with same colors as lines represent the percentage of most diverse biological replicates found for each one of the biological replicates. (A) Samples pre-incubated in the light; (B) samples pre-incubated in the dark.
