# Supplementary material for: Isolation of a significant fraction of non-phototroph diversity from a desert Biological Soil Crust
Source: Front Microbiol. 2015 Apr 14;6:277. doi: 10.3389/fmicb.2015.00277 (PMC4396413; doi:10.3389/fmicb.2015.00277)
Supplement: Supplementary file 7 [file Image3.PDF]

Supplementary Figure 3

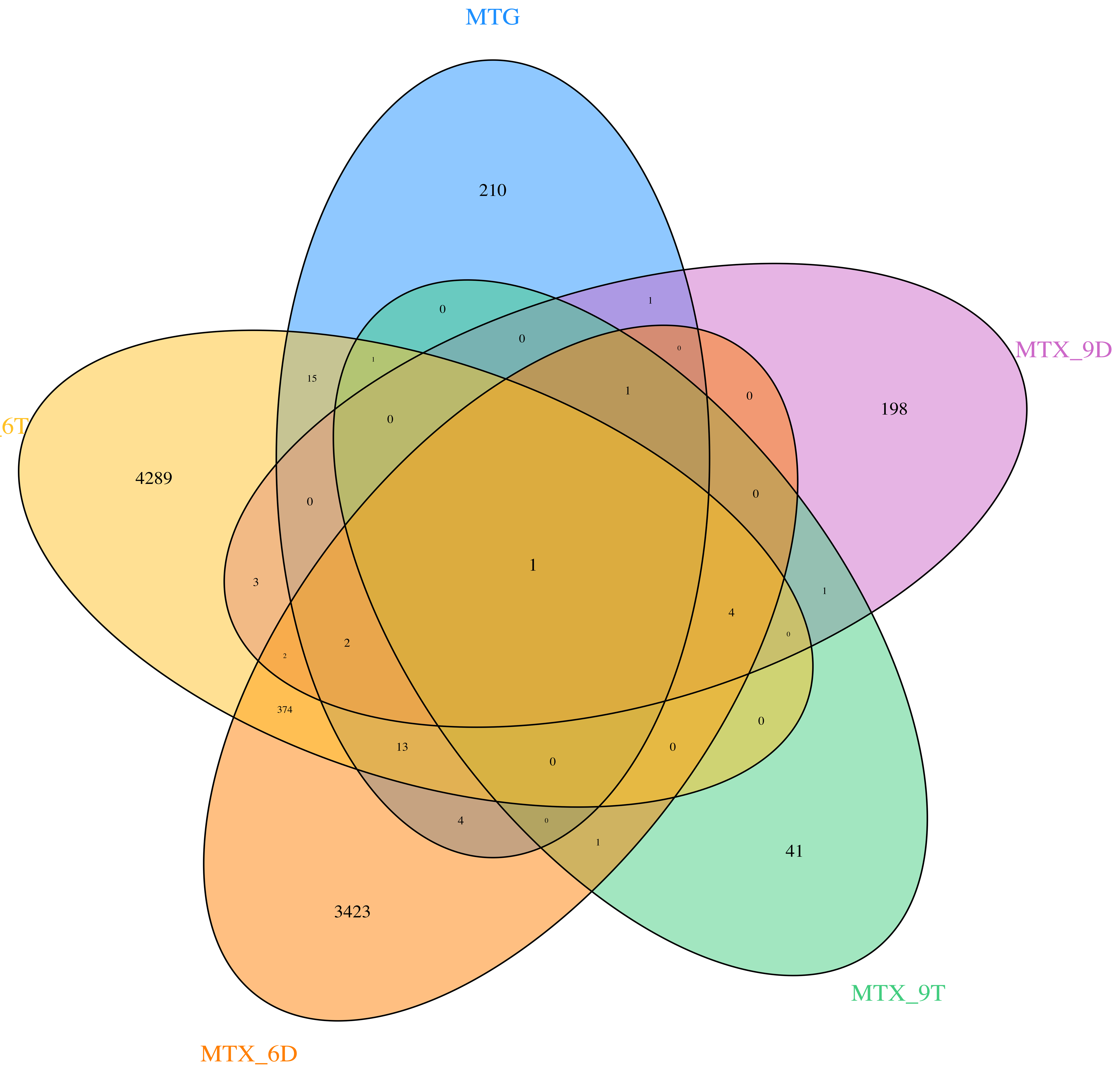

Figure Legend. Venn diagrams showing the intersection of EMIRGE reconstructed SSU of the rRNA gene clustered at 97% similarity from the different libraries. MTG, metagenome library constructed with DNA extracted prior wet-up; MTX_6T, metatranscriptome library from night time (18h after wet-up) sample constructed with total cDNA; MTX_6D metatranscriptome library from night time (18h after wet-up) sample reconstructed with double strand nuclease (DSN) treated cDNA; MTX_9T, metatranscriptome library from day time (25.5h after wet-up) constructed with total cDNA; MTX_9D, metatranscriptomic library from day time (25.5h after wet-up) constructed with double strand nuclease (DSN) treated cDNA.
