# Supplementary material for: Isolation of a significant fraction of non-phototroph diversity from a desert Biological Soil Crust
Source: Front Microbiol. 2015 Apr 14;6:277. doi: 10.3389/fmicb.2015.00277 (PMC4396413; doi:10.3389/fmicb.2015.00277)
Supplement: Supplementary file 8 [file Image4.PDF]

Supplementary Figure 4

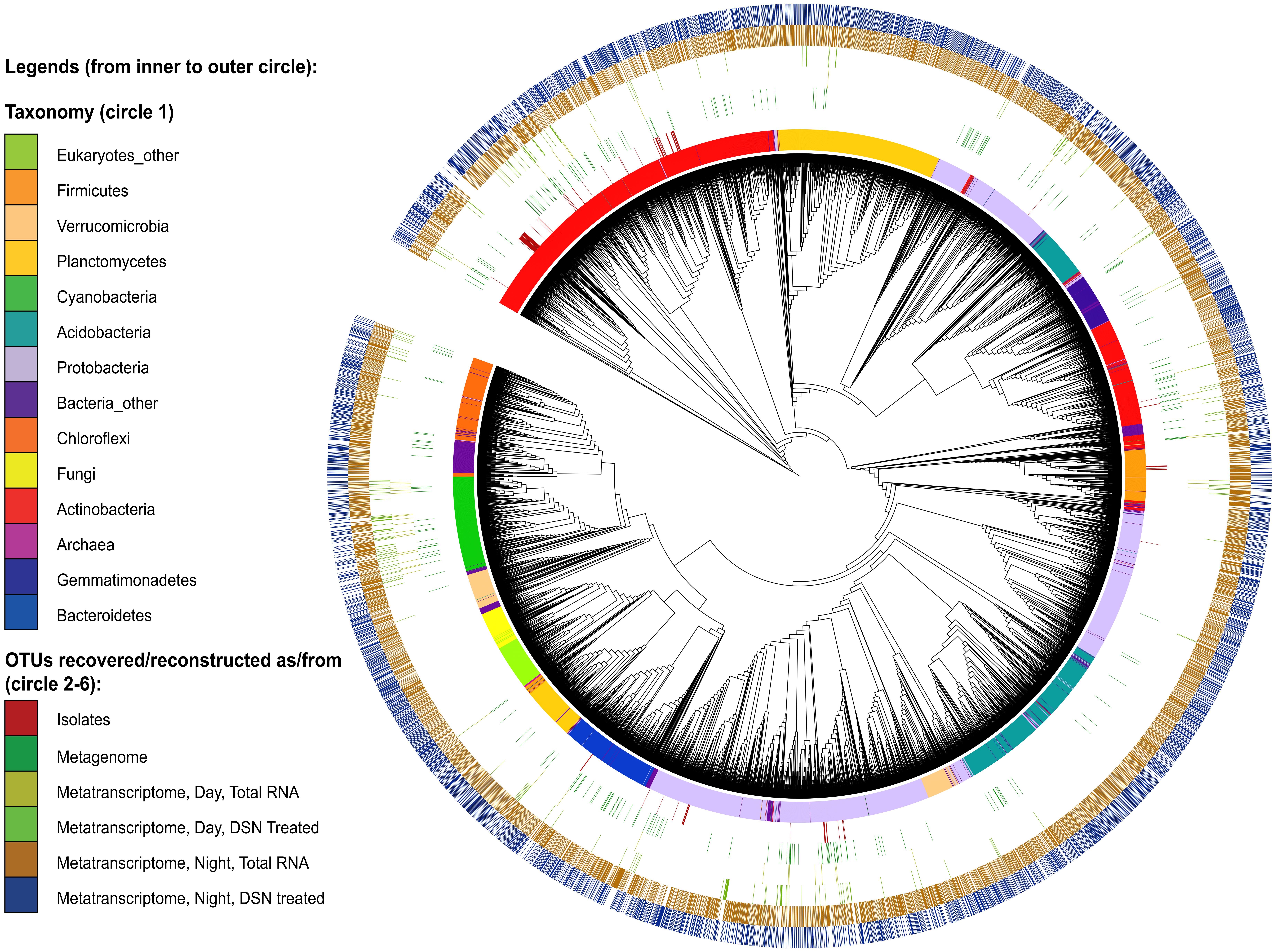

Figure Legend. Phylogenetic tree with EMIRGE reconstructed SSU rRNA genes from the Biological Soil Crust and sequences from isolates, clustered at 97% similarity. Datasets rings, from the inner to the outer circles, represent: Dataset Taxonomy, taxonomy of EMIRGE reconstructed SSU rRNA genes from the Biological Soil Crust and from isolates; Dataset Isolates, representative isolates; Dataset Metagenome, EMIRGE reconstructed SSU of the rRNA gene from the metagenome library; Dataset Metatranscriptome Night Total RNA, EMIRGE reconstructed SSU of the rRNA gene from night time (18h after wet-up) metatranscriptome constructed with total cDNA; Dataset Metatranscriptome Night DSN treatment RNA, EMIRGE reconstructed SSU of the rRNA gene from night time (18h after wet-up) metatranscriptome constructed with cDNA treated with double strand nuclease; Dataset Metatranscriptome Day Total RNA, EMIRGE reconstructed SSU of the rRNA gene from day time (25.5h after wet-up) metatranscriptome constructed with total cDNA; Dataset Metatranscriptome Day DSN treatment RNA, EMIRGE reconstructed SSU of the rRNA gene from day time (25.5 after wet-up) metatranscriptome constructed with cDNA treated with double strand nuclease.
